# Room-Temperature Fluorescence Lifetime of Pseudoisocyanine (PIC) J Excitons with Various Aggregate Morphologies in Relation to Microcavity Polariton Formation

**DOI:** 10.3390/ijms13055851

**Published:** 2012-05-15

**Authors:** Yuki Obara, Keita Saitoh, Masaru Oda, Toshiro Tani

**Affiliations:** 1 Graduate School of Engineering, Department of Applied Physics, Tokyo University of Agriculture and Technology, 2-24-16 Naka-cho, Kogane-i, Tokyo 184-8588, Japan; E-Mails: 50009834102@st.tuat.ac.jp (Y.O.); keita.saito@konicaminolta.jp (K.S.); odamasa@cc.tuat.ac.jp (M.O.); 2 Division of Advanced Applied Physics, Institute of Technology, Tokyo University of Agriculture and Technology, 2-24-16 Naka-cho, Kogane-i, Tokyo 184-8588, Japan

**Keywords:** J aggregates, PIC-Cl, fluorescence decay time, fluorescence quantum efficiency, fibril-shaped macro-aggregates, Frenkel exciton, cavity polariton, dephasing

## Abstract

The results of room-temperature fluorescence lifetime measurements are reported for the excitation of J aggregates (Js) of pseudoisocyanine chloride (PIC-Cl) prepared in potassium polyvinyl sulfate (PVS) polymer thin films, their aqueous solutions, and NaCl aqueous solutions. Variations of the microscopic morphologies of the aggregates were investigated. The results show that fluorescence decay features correlated to the morphology change. The observed fluorescence lifetime and quantum efficiency of PIC J aggregates (PIC-Js) in a NaCl aqueous solution were 310 ps and 28%, respectively. The lifetime of the fibril-shaped macroaggregates prepared in PVS thin films was below the instrumental time resolution of 5 ps, and the efficiency decreased to below 3%. The results indicate that PIC-Js prepared with PVS polymers have an increased nonradiative contribution to the excitation deactivation process. In particular, macro-Js with isolated fibril-shaped structures revealed nonradiative pathway(s) that are closely associated to the specific packaging morphology of the constituent meso-Js. The possibility of a destructive effect on the formation of cavity-polaritons is also discussed.

## 1. Introduction

Dynamic processes of Frenkel excitons in self-assembled molecular J aggregates (Js) have been intensively investigated over the last few decades. They possess unique optical responses that differ from both the monomeric constituents and bulk crystals. These characteristics originate from the strong coupling between the transition dipoles of adjacent molecules [1–5]. It is also notable that Js are nanomaterials and have low dimensionality. This type of aggregates include not only those showing simple J or H aggregate features [6,7] but also those exhibiting Davydov splitting [8] and even cyclic complexes [9–11]. We have investigated the optical and excitonic properties of pseudoisocyanine (PIC) Js (PIC-Js) as a prime prototype, prepared in polyanionic polyvinyl sulfate (PVS) polymer matrices [12–14] in which significant discrepancies remain. PIC-Js possess complex J-band structure mostly due to the presence of more than one molecule per unit cell. The overlapping of the monomer bands to these higher J-bands further complicates the issues. Currently, we are also concerned with the formation of exciton-photon strong coupling at room temperature [15,16].

Planar quantum microcavities (QMCs), which contain Js as active media, are gaining increased attention. This regime is identified by the observation of anti-crossing behaviors between the cavity-photon and J-exciton dispersion curves near their resonance energy; the minimum energy corresponds to the vacuum Rabi splitting (2Δ). This normal mode is referred to as a cavity-polariton, and 2Δ is a measure of the exciton-photon coupling strength. It is significant that, due to the large oscillator strengths of the Js, strong coupling can be easily achieved even at room temperature [17–19], while lower temperatures are required for inorganic semiconductors [20,21].

In our previous work, well-defined cavity–polariton features were observed in the QMC structures containing PIC-Js as the active media, even in a fairly low-Q metal-mirror cavity at room temperature. We confirmed that the observed 2Δ, which easily exceeded more than 150 meV, increased with increasing J concentration [16], as predicted theoretically [19,20]. It is not easy to control the concentration of Js or even their shape and size in an actual device structure. However, it is important, not only for applications but also from a basic photo-physical point of view, to understand cavity-polariton behavior at the high-density limit of the Js. Careful study of the light-matter interaction for varying Js density provides information on the inter-J-chain as well as the intra-J-chain interactions in the unique molecular system.

In the case of PIC-Js prepared in anionic polyelectrolyte PVS polymer films, isolated fibril-shaped macroaggregate structures are easily obtained [22–26]. These structures are presumably bundles of multiple, almost parallel, constituent mesoaggregates. They are highly suitable candidates for QMCs because the density of the active material is near the limit. Based on our previous observations of the concentration dependence, we expect that the 2Δ of the single isolated fibril-shaped Js incorporated in the QMC structures will exceed 250 meV, the largest value recorded to date [16].

However, preliminary results did not meet expectations; no definitive manifestation of cavity-polariton formation has been observed [27]. This phenomenon was confirmed in our present work using reformulated samples. This finding is significant because in solid films of Js, prepared by spin-coating a saturated solution of a specific dye salt such as (PIC^+^)_2_B_10_H_10_^2−^, cavity–polariton formation was detected at room temperature with a 2Δ of ~50 meV [28–30].

In order to tackle the anomalous cavity-polariton features, we must consider the dynamic properties of the Frenkel exciton in PIC-Js prepared in PVS matrices with respect to their microscopic morphologies. The morphologies will include not only the J-formation catalytic reagents but also inter-mesoaggregate conformations and their local environments as well. Preliminary results of fluorescence decay in various types of macroaggregates in PVS-based matrices and in saline aqueous solutions are presented here.

## 2. Experimental

**Figure 1:**
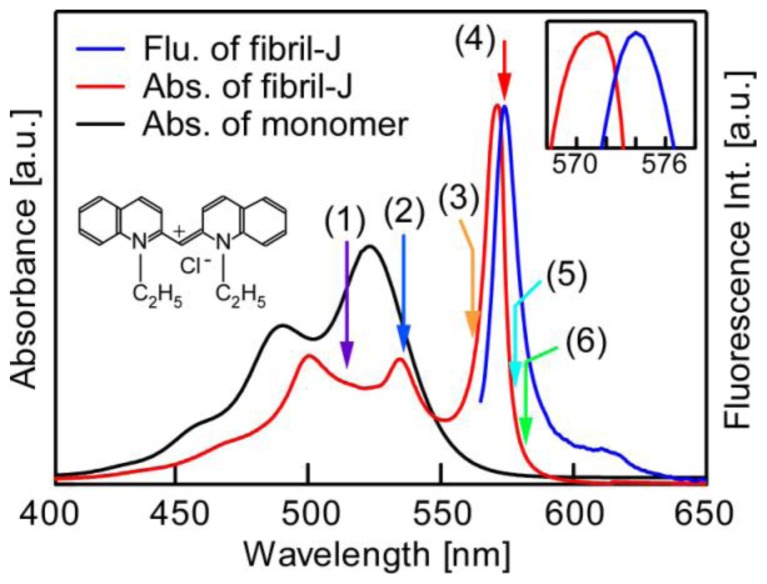
Macroscopic absorption (red) and fluorescence (blue) spectra of fibril-shaped pseudoisocyanine chloride (PIC-Cl) (inset) Js at room temperature. The former was obtained in the thin-film sample of a potassium polyvinyl sulfate (PVS) matrix and the latter in a PVS dissolved aqueous solution, respectively. The absorption spectrum of PIC monomers is also shown (black) for comparison. The inset in the right-hand side shows the existence of a distinctive Stokes shift of 2.8 ± 0.3 nm. The numbers on the absorption line indicate excitation wavelengths for the fluorescence decay profiles (see Figure 3).

Pseudoisocyanine chloride dye (1,1′-diethy-2,2′-cyanine chloride, PIC-Cl, Hayashibara Biochem. Labs, Inc.) was used without further purification. Since its introduction, PIC has been well known for its remarkable J formation [1–5]. In Figure 1, typical room-temperature bulk absorption and emission spectra are shown for samples prepared in the polymeric media, as described below. The monomer absorption spectrum is also shown for comparison.

First, Js were grown in a thin film of potassium polyvinyl sulfate (PVS–K). PVS–K is also used as a part of the active media in QMCs. The PVS films were fabricated directly on the surface of silica glass substrates or on evaporated Ag bottom mirrors by spin-coating an aqueous solution at an elevated temperature of 95 °C. To form flat layers, the substrate was set off-center from the rotation axis and coated with a two-step rotation at 600 rpm for the first 5 s followed by 3000 rpm for the next 30 s.

The basic procedure for J preparation is similar to those described elsewhere [12–16]. An aliquot of 0.4 mL of PIC-Cl methanol solution was added to 3.6 mL of PVS-K aqueous solution (3.6 mg·mL^−1^).

For the films with dispersive Js, the initial concentration of PVS–K solution was increased to 26 mg·mL^−1^. This procedure is commonly used to fabricate QMC samples due to its ease of reproducibility. The sample morphologies were determined from the overall texture of topographical images produced by an optical far-field microscope [24,25]. When possible, these images were cross-referenced with local reflection and fluorescence spectra for further verification.

Liquid PVS-K samples were prepared in a similar manner. An aliquot of the spin-coated sample mentioned above was transferred to a silica glass cell with a 0.1-mm optical path length.

For saline aqueous solutions, the samples were prepared by mixing 0.2 mL of PIC–Cl methanol solution (1.5 × 10^−3^ mol·L^−1^) in a 5.8 mL NaCl solution (5.0 mol·L^−1^; 99.5% distilled water; *ρ* ≥ 18 MΩ·cm). The final PIC dye concentration was 5.0 × 10^−5^ mol·L^−1^, which is almost two orders of magnitude less than previously reported concentrations [31,32]. A silica glass cell with a 1-mm optical path length was used for observations. The cell was equipped with a thin stirrer.

For planar QMC structures, vacuum Ag deposition for upper and lower mirror formation was introduced between Js procedures. First, in the case of dispersed Js, the bottom mirror was evaporated onto a silica glass substrate. The active layer was then fabricated by spin-coating with a PVS aqueous solution having a specific PIC-J concentration. The film thicknesses coincided with the half-wavelength (λ/2) thicknesses to form the cavity, which allowed the photons to resonate with the exciton energy at a specific incident angle. Once the top surface of the prepared layers was sufficiently smooth and flat, the top mirror was fabricated by successive vacuum Ag deposition. The thickness of the top mirror was ca. 30 nm. All the QMC samples contain the Js as active media.

**Figure 2:**
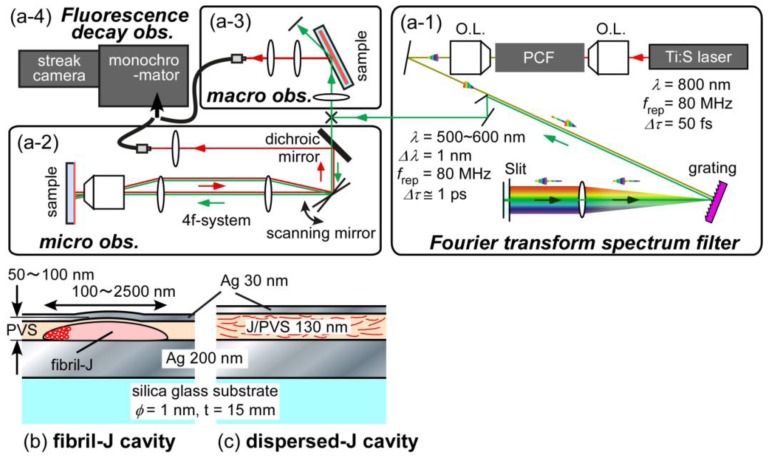
Schematic of the optical setup (**a**) for the observation of fluorescence decay profiles and microcavity sample structures (**b**, **c**). (a–1) Fourier transform spectral filter with photonic crystal fiber (PCF) for continuous white light generation and mode-locked Ti:Sapphire Laser provide ca. 1-ps excitation light pulses with a 1-nm wavelength width. (a–2) For microscopic decay observation of single fibrils, we used scanning microscope optics. (a–3) For ordinary macroscopic observation, conventional optics was utilized. (a–4) Decay signals were detected with a Streak camera through a monochromator (*f* = 300 mm). Microcavity structures with Ag metal mirrors are shown schematically: (**b**) fibril-shaped and (**c**) ordinary dispersed Js as active media prepared with PVS thin film matrices. The cross-sectional view of the fibril is shown.

The procedure for fibril-shaped Js formation requires that the thickness of each fibril is on the order of a few tens of nanometers to a hundred nanometers, the width is on the order of micrometers, much larger than the thickness, and the aspect ratios are roughly several tens or more. Fibril-type QMCs are fabricated by Ag vacuum deposition of top mirrors onto the single fibrils, which already have PVS on the bottom mirror, as described above. The top surfaces were checked to ensure surface smoothness and flatness for incident light introduction. The morphology and size of the fibrils were monitored by atomic force microscopy (AFM) (Nanocute, SII NanoTech, Inc.). Schematic diagrams of the two types of QMCs are shown in Figure 2(b,c).

In planer QMC experiments, the cavity mode was tuned by varying the incident angle of illuminating light [20], which corresponds to varying the in-plane component of the mode wavenumber vector. We measured angle-resolved reflection spectra by scanning the incident angle in the vicinity of the cavity photon energy corresponding to the resonance energy required for PIC-J exciton coupling. For analysis of both fibril-shaped and dispersed-J QMC samples, we developed a microscope optical setup with a sub-wavelength spatial resolution [33], less than the geometrical width of a single fibril.

To study fluorescence decay, optical pulse generation and detection require a time resolution on the order of several picoseconds for this experiment. Also, to coordinate the optics with the microscope system and achieve spectral resolution suitable for the J band, we customized our optical system as shown in Figure 2(a). To excite the samples, optical short pulses of ca. 1 ps and ca. 1 nm spectral width were applied with a mode-locked Ti:Sapphire laser (KM Labs Inc., Grifin–80M, Δ*τ* = 50 fs). For continuous white light generation, we used a 1.8-μm core-diameter photonic crystal fiber (PCF; NKT Photonics, FemtoWHITE 800) (a–1). For wavelength selection, a Fourier transform spectrum filter (FTSF) was used [34]. The excitation pulses were then introduced into (a–2) microscope local observation optics or (a–3) macroscopic bulk observation optics, separately. The pulse intensity was roughly 2 × 10^6^ photons/pulse. The signal light was then focused into large core-diameter optical fibers and detected by a streak camera (Hamamatsu Photonics K.K., C4334) through a monochromator with a 300 groove/mm grating (Acton Research Corp., SpectraPro-300i). The instrumental response functions were on the order of ca. 40 ps. The final time resolution of the convolution fitting was ca. 5 ps with a spectral resolution of ca. 0.5 nm.

## 3. Results and Discussion

**Figure 3:**
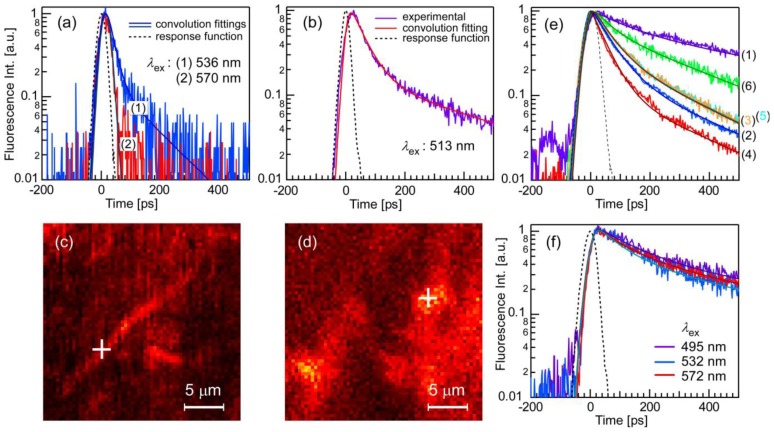
Fluorescence decay profiles of PIC-Cl J aggregates with various microscopic environments at room temperature. (**a**) and (**b**) Decay profiles from the diffraction-limited local area (ca. 0.3 μm in diameter) of distinct fibril-shaped single macroaggregates and where the formation of such bundle-like structures seems insufficient, respectively. Numbers in (a) show the differences with respect to the excitation wavelength change; (**c**) and (**d**) Topographic imaging by scanning far-field fluorescence microscopy (see Figure 2(a–2)), indicating the aggregation morphologies at locations corresponding to (a) and (b), respectively. The two positions are different locations within the same sample, prepared in a ***PVS thin-film matrix***. The crosses (+) indicate the locations of the data acquisition points; (**e**) Decay profiles of fibril-shaped Js in ***PVS aqueous solutions*** measured with the macroscopic optical setup. The numbers indicate the difference in the excitation wavelengths, corresponding to those in Figure 1; (**f**) Decay profiles of PIC-Cl Js prepared in ***NaCl aqueous solution***. The macroscopic optical setup was used. The broken lines represent instrumental response functions. The solid lines show convolution fitting, from which the decay times were deduced.

We observed room-temperature fluorescence decay profiles of PIC-Js prepared with anionic polyelectrolyte PVS polymers with various macroaggregate morphologies, *i.e.*, in PVS polymer thin films and in their aqueous solutions. We also measured the Js prepared in NaCl aqueous solution as a standard. The results (Figure 3) indicate a clear correlation to the macroaggregate morphologies.

Figure 3(a) shows typical decay profiles of single fibril-shaped macro-J aggregate observed with scanning far-field microscope optics, as shown in Figure 2(a–2). The excitation pulse intensity was ca. 3 × 10^11^ photon/(pulse cm^2^) at both (1) λ_ex_ = 536 nm and (2) λ_ex_ = 570 nm. Figure 3(c) shows a topographic fluorescence image observed simultaneously using the microscope shown in Figure 2(a–2) [35], where the lifetime-monitored location is indicated by a cross (+). The spot size, *i.e.*, the spatial resolution of the objective, was ca. 0.3 μm in diameter. The decay feature excited at λ_ex_ = 536 nm shows double-exponential kinetics; the faster and slower decay times were 19 ps (66%) and 121 ps (34%), respectively. Basically similar features are obtained over the distinctive fibril regions. For λ_ex_ = 570 nm, the nearly single-exponential fit resolves a decay time of 5 ps. The 5-ps decay time corresponds to the time resolution of the experiment, thus indicating that the decay time in this case was below 5 ps. As for the fluorescence quantum efficiency *Φ*, it is not an easy task for the single fibril-shaped macroaggregated structures to measure by the microscope. However, we tried to estimate it by comparing with that of Rhodamine B; *Φ* at λ_ex_ = 536 nm is roughly 3% or less. This is, so to speak, an upper limitation in the present S/N ratio and very low.

Currently, the origin of the nonexponential features, such as the exciton annihilation process, is not clear [26,36,37]. In fact, we have not yet determined definitively the excitation intensity dependences, mainly due to the fairly strong photodegradation of the samples [32].

Figure 3(b) shows the fluorescence decay profile of the same sample at a different location (λ_ex_ = 513 nm, pulse intensity; 2 × 10^11^ photon/(pulse cm^2^). As shown in the topographic image of Figure 3(d), the morphology of the sample at or around the observed point (+) seems to consist of random networks of entangled macroaggregates, which is different from the fibril structure shown in Figure 3(c). Compared to the decay profile of Figure 3(a–1), *i.e.*, off-resonance excitation, the decay times increased. In fact, double-exponential convolution is required to fit the profiles, resulting in decay times of *τ*_1_ = 45 ps (50%) and *τ*_2_ = 390 ps (50%). This suggests the formation of an intermediate aggregate form, one in between the fibril-shaped bundled macroaggregates and those in the PVS aqueous solutions described below.

Figure 3(e) shows the decay profiles of PIC-Js prepared in a PVS aqueous solution with various excitation wavelengths. The monitored sample area was ca. 2 mm in diameter, and a 100-μm optical path length was used (cell thickness: 1.25 mm). The pulse intensities were rather low, on the order of ca. 8 × 10^7^ photon/(pulse cm^2^), because the sample was susceptible to photodegradation; flow-type cells were not an option for this sample. As shown in Figure 3(e), the decay kinetics are represented by a double-exponential profile. While there appears to be distinct excitation wavelength dependence, the decay times increased in PVS aqueous solutions compared to PVS films. The decay times obtained with double-exponential convolution fitting are summarized in the Table 1. The highest decay rate seems to correspond to resonance excitations of the J band. The origin of these excitation wavelength dependences is not yet evident; however, it means basically the emission is not coming from the lowest exciton band and seems distinctive to the aggregates formed with anionic polyelectrolyte PVS polymers. Another possibility is that this may exhibit high decay rates for excitation wavelengths meeting high oscillator strength and vice versa.

It should be noted here that, while there is a lot of light coming from the places between large aggregates, as is seen in Figure 3(c,d), these should not be ascribed to the monomers. We have obtained no detectable monomer contributions in the fluorescence spectra yet; they are from dispersed meso-J aggregates [12–14,24,25].

To gain insight into this process, we also measured the fluorescence lifetime of PIC-Js prepared in a NaCl aqueous solution. The result is shown in Figure 3(f). The decay kinetics tends to nearly mono-exponential and the λ_ex_ dependence almost disappears. However, apparently it still remains. The decay times are obtained with double-exponential fittings and the results are summarized in the Table 2. We basically consider the shorter decay-time component as being decreased here. On average, by integrating all the accumulation time for near resonance excitation, the fluorescence lifetime of the PIC-Js prepared in NaCl aqueous solution was around *τ*_f_ = 310 ps, which is in good agreement with previous studies in aqueous solutions [38].

For PIC-Js in NaCl aqueous solution, we also evaluated the fluorescence quantum yield, using Rhodamine B in ethanol solution (2.1 × 10^−5^ mol·L^−1^, base form) as a standard reference. In general, there exist fundamental difficulties in determining the quantum efficiencies of Js [39]; however, we tried to make a rough estimate. The obtained quantum yield *Φ* with off-resonance excitation was ca. 28% on average. Using the relationship *Φ* = *τ*_f_/*τ*_0J_, the radiative lifetime of the PIC-Js in NaCl aqueous solution can be estimated as *τ*_0J_ = 1.1 ns at room temperature.

If we take 3.7 ns as the radiative lifetime, *τ*_0_, of PIC monomers [40], the simple relation *τ*_0J_ = *τ*_0_/*N*_del_ [41–43] provides us with the number *N*_del_ of monomeric molecules in the PIC-Js is ~3.4. Thus, the excitation is delocalized coherently in approximately 3 or 4 monomer size, and *N*_del_ is actually larger than unity even at room temperature. This number is in good agreement with previous studies [31]. For the *N*_del_ of PIC-Js grown in the glass matrix of water/ethylene glycol, *N*_del_ seemed to decrease to 3 or 4 already at around 140 K [8,44,45]. Hence, we propose that the electron–phonon coupling in PIC-Js prepared in NaCl aqueous solution must be less than the coupling in Js grown in the solvent. Actually, the formation of rod-like structures with a cross-sectional diameter of 2.3 nm is suggested for NaCl aqueous solutions [46,47]. It should also be noted that the present sample was prepared with a reduced dye concentration (by a factor of 20), while the NaCl concentration was increased by a factor of 10. As a result, the rod-like structures were sufficiently diluted in the solution and separated sufficiently from each other.

The oscillator strength of the PIC-Js prepared in the PVS aqueous solution was evaluated based on the absorption spectra and compared to that of the PIC monomers in methanol solution. The result indicates an excitation delocalization number of *N*_del_~9 at room temperature. The agreement within one order of magnitude is rather amazing if we consider that the molecular orientation of Js is totally neglected in the latter evaluation.

Based on these fundamental findings of PIC-Js in various environmental conditions, we tentatively reached the following conclusions. The excitation wavelength dependence and the appearance of short lifetime components should be inherent to PIC-Js prepared with anionic polyelectrolyte PVS polymers [26]. Furthermore, in the case of fibril-shaped macroaggregate structures, in which the portion of almost parallel and closely-packed constituent mesoaggregates approaches the maximum, the decay rate of resonance excitation approaches the 5-ps time resolution of the experiment. The appearance of the shorter decay-time components may be a manifestation of nonradiative pathway(s) closely associated with such specific structures.

We briefly describe the typical cavity-polariton features of the QMC samples in which PIC-Js are used as the active media in both the dispersed-J form and in the fibril-shaped J form, as shown in Figure 2(b,c), respectively. Both were reinvestigated on newly prepared samples using angle-resolved microscopy; the details are described elsewhere [15,16].

**Figure 4:**
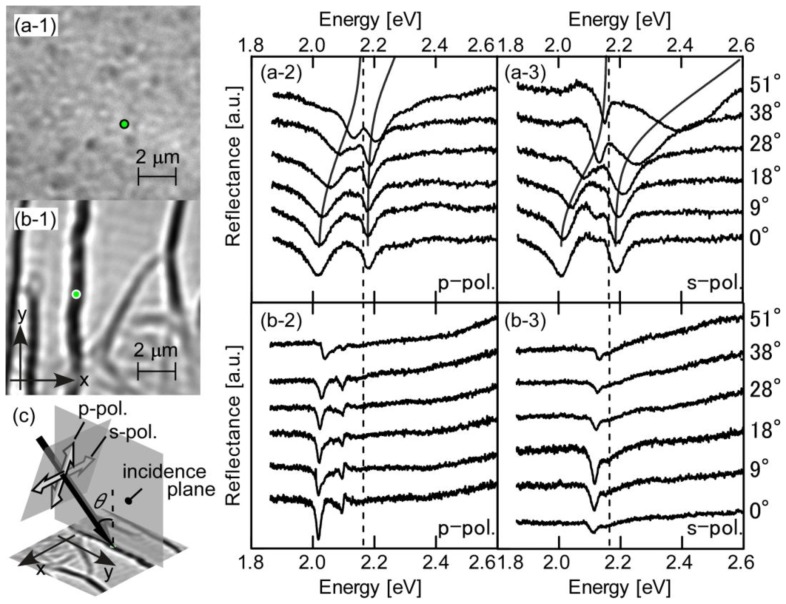
Typical examples of angle-resolved microscopic reflection observations for samples with (**a**) dispersed Js and (**b**) fibril-shaped Js, respectively, prepared as active layers in Ag–Ag mirror microcavity structures (see Figure 2(c,b)). (a–1) Topographic reflection microscope image of the local sample surface. The green circle indicates the size and location of the observed spot. (a–2) and (a–3) show angle-resolved local reflectivity spectra obtained with p-polarized and s-polarized incident light, respectively. The broken lines indicate the exciton energy of Js, ca. 2.16 eV and assumed constant here. The same descriptions hold for (b–1), (b–2), and (b–3), respectively; (**c**) Relative configurations of the incident light and its polarization to the sample surface.

Figure 4 shows typical local reflection spectra for (a) dispersed Js and (b) fibril-shaped Js and the corresponding topographic reflection images of the QMC sample surfaces. The green spots in Figure 4(a–1) and 4(b–1) indicate the local area that was actually monitored. The images are photographed with an oil-immersion objective (Nikon, Plan Apo TIRF100×, N.A. = 1.45) [16]. As shown in the figure, the spatial resolution of the objective was sufficiently small to deduce the localized spectroscopic information of a single fibril. Figure 4(c) describes the experimental configuration of the sample and incident light polarizations.

For dispersed-J samples (Figure 4(a–2) and 4(a–3)) distinct anti-crossing behavior was clearly observed in the vicinity of resonance for both polarizations, even at room temperature. These features indicate that in PVS thin films with dispersed Js, Frenkel excitons, *E*_ex_, and cavity photons, *E*_ph_(*θ*), are interacting coherently, eventually forming cavity-polaritons as they reach the strong-coupling regime. The upper and lower polariton branches can be fit with a simple dispersion relation (solid curves) [20,48]: *E*_U,L_ = (*E*_ex_ + *E*_ph_(*θ*))/2 ± [(*E*_ex_ − *E*_ph_(*θ*))^2^ + (2Δ)^2^]^1/2^/2.

The obtained vacuum Rabi splittings, 2Δ, were 78 meV at *θ* = 47° for p-polarization and 121 meV at *θ* = 31° for s-polarization. Here Δ is the interaction energy between the exciton and cavity-photon and is proportional to the square root of the oscillator strength of the exciton transition. This fact is quite remarkable, as previously noted [15,49,50], considering that this performance was obtained using rather low-Q (~40) Ag-mirror cavities. The potential of organic semiconductors and Frenkel excitons is evident. The observation of polariton features in both p- and s-polarized configurations indicates that excitons acting as cavity–polaritons in the Js are, on the nanometer scale, far less than the wavelength of light. On the other hand, the features observed for fibril-shaped Js are quite different. Figure 4(b–1) shows a topographic microscope reflection image of single fibrils in the QMC, photographed through the top Ag mirror, ca. 30 nm thick. The thin semi-transparent Ag top mirror allows sufficient access for identification of the individual fibrils inside the cavity. While the imag e was obtained with nonpolarized light illumination, the transition dipole of the J band is oriented in the direction of the long axis of the fibril [12,13], *i.e.*, in the direction of the exciton wavenumber vectors [22,23].

Angle-resolved local reflectivity spectra are shown in Figure 4(b–2) and 4(b–3). As can be seen in the figures, dip structures appeared with almost no angular dependence on the tuning of the incident light. For p-polarization, small narrow dips appeared at or around 60 or 150 meV, lower than *E*_ex_ (broken line). The p-polarization possesses the in-plane component of the cavity–photon wave vector and the electric field component of the incident light, both parallel to the long axis of the fibrils. Both p- and s-polarizations are demonstrated here; however, the appearance of such a dip structure in s-polarization depends on the samples, and in some cases, is not observed [16], see also Figure 11 in [51]. Typical features, such as rather sharp dip(s) and insensitivity to the tuning of the angle of incident light, are similar, but the strong coupling characteristics seem to disappear for fibril-shaped Js. This result is far from our initial expectation.

The cause of this anomaly is not clear at present and, as stated earlier, motivated the present work. In the following, we present key factors to consider with regard to the decay lifetime measurements.

First, it is worth noting the effect of an abrupt increase in the refractive index *n* of the active layers in the vicinity of exciton resonance. The J–band is a result of very intense oscillator strength concentration over a narrow spectral range. For dispersed Js, *n* is near that of the PVS polymer film, ~1.4, and remains almost constant because of its larger volume fraction in the active layers. On the other hand, for fibril-shaped Js, in which strings of meso-Js are packed to the highest density limit, *n* can deviate from that of the dispersed Js [22,23]. While a quantitative estimate of *n* for single fibrils is not definitive at this moment, *n* on the order of 3 is sufficient to form realistic *λ*/2 cavities with fibril thicknesses of ~50–100 nm, as shown in Figure 2(b).

An abrupt increase in *n* may also affect the measurements. We have been accumulating experimental data carefully with various sample conditions, taking careful note of sample thickness and shape; however, there has been no indication of such anomalous behavior to date.

One possible experimental issue is the shape of the Ag top mirror comprising the QMC (see Figure 2(b)). While the effect of the Ag-mirror curvature is sufficiently small under simulation conditions, we never have encountered in the huge amount of accumulated samples with various fibril conditions yet. Future experiments will address the use of totally flat Ag or DBR mirrors.

Another possible interpretation is that only the lower cavity-polariton branch appears within our observation energy window due to the realization of exceptionally strong exciton-photon coupling [52]. This possibility is, however, not realistic if we take into account that the solid J-aggregate films were prepared from (PIC^+^)_2_B_10_H_10_^2−^ [28–30]. We also suppose that the observed shift from *E*_ex_ is too small in this case. Again, we have not yet observed even a trace existence of the upper branch(s).

Another interpretation is that the strong coupling regime experienced interference in the case of fibril-shaped Js. The fluorescence decay characteristics of the fibril-shaped PIC-Js described above may indicate that such destruction of coherence in strong exciton–photon coupling may be possible if energy transfer, exciton migration, or other dephasing mechanisms occur within the macroaggregate structure. Such mechanisms are certainly a possibility if the distance between the exciton segments is reduced in fibril-shaped macroaggregates [53–57]. The observed fluorescence lifetime in this case reached less than 5 ps.

We have previously reported the appearance of remarkable band broadening with dip structures in local reflectance spectra for thick fibril-shaped Js [58], appearing in single fibrils without Ag mirrors. This broadening and dip appearance can be interpreted by the macroscopic theory for a dielectric slab [58,59]. If the coherence of the cavity–polariton in the fibril-shaped Js is compromised, similar mechanisms may manifest themselves as the anomaly. Further investigations on exciton dynamics are required.

## 4. Conclusion

We have presented fluorescence lifetime observations for PIC-Js prepared with PVS polymers with various morphologies. The PIC-Js prepared with PVS polymers have an increased nonradiative contribution to the excitation deactivation process. In particular, macro-Js with isolated fibril-shaped structures revealed nonradiative pathway(s) that are closely associated to the specific packaging morphology of the constituent meso-Js. The results are discussed in relation to the strong coupling features of the PIC-J Frenkel excitons at room temperature. Many questions remain, and further investigations into the dephasing are underway.

## Figures and Tables

**Table 1:** Excitation wavelength λ_ex_ difference in the fluorescence decay times of PIC-Js prepared in the PVS aqueous solution. *τ*_1_ and *τ*_2_ are the shorter and longer decay times, obtained by the double exponential convolution fittings, respectively. Ratios are the integrated existence ratio of each component.

*λ*_ex_ [nm]	*τ*_1_ [ps] (ratio_1_ [%])	*τ*_2_ [ps] (ratio_2_ [%])

511	110 (12)	660 (88)
534	55 (59)	270 (41)
560	49 (36)	220 (64)
572	42 (70)	260 (30)
576	56 (43)	250 (57)
580	55 (17)	310 (83)

**Table 2:** Excitation wavelength differences in the fluorescence decay times of PIC-Js prepared in the NaCl aqueous solution. Definitions of each heading are the same as in the Table 1.

*λ*_ex_ [nm]	*τ*_1_ [ps] (ratio_1_ [%])	*τ*_2_ [ps] (ratio_2_ [%])

495	100 (16)	560 (84)
532	86 (23)	540 (77)
572	89 (17)	520 (83)
